# Supplementary material for: A whole-animal phenotypic drug screen identifies suppressors of atherogenic lipoproteins
Source: eLife. 2026 Aug 27;14:RP105314. doi: 10.7554/eLife.105314 (PMC13521566; doi:10.7554/eLife.105314)
Supplement: Supplementary file 2 [file elife-105314-code1.zip › JHDL_primary_result/NADIDE/NADIDE_JHDL_4_020_020_2019_05_10_11_37.pdf]

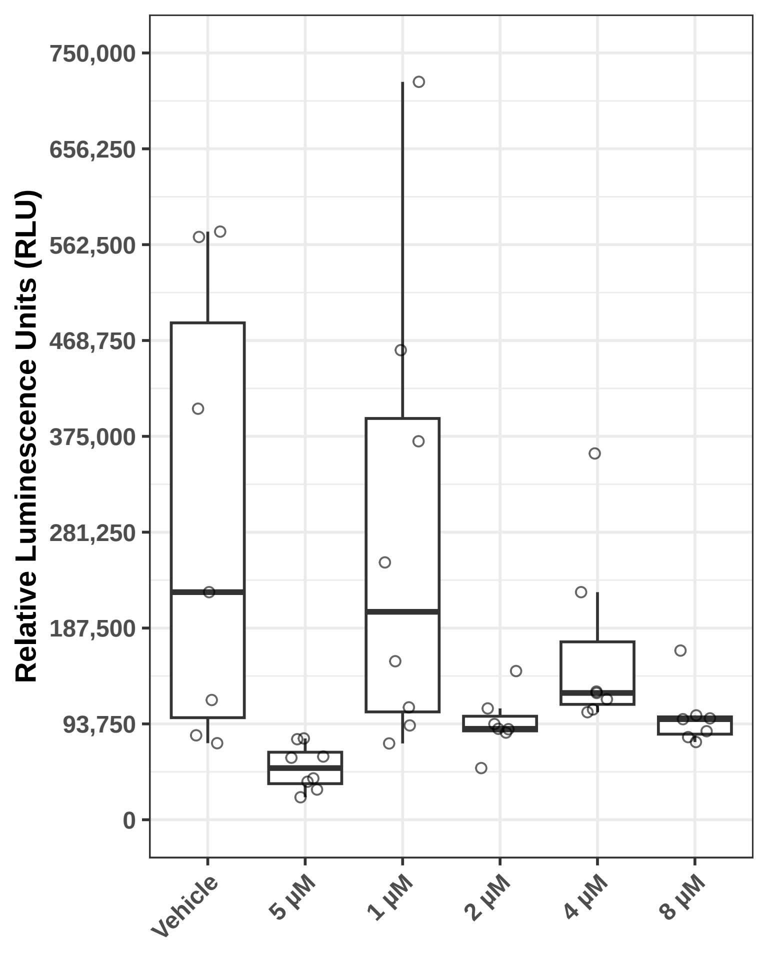
Relative Luminescence Units (RLU)
750,000
656,250
562,500
468,750
375,000
281,250
187,500
93,750
0
Vehicle
5 µM
1 µM
2 µM
4 µM
8 µM
